# Identification of heat shock factor binding protein in *Plasmodium falciparum*

**DOI:** 10.1186/1475-2875-13-118

**Published:** 2014-03-27

**Authors:** Syed K Sayeed, Varun Shah, Shweta Chaubey, Meetali Singh, Shuba V Alampalli, Utpal S Tatu

**Affiliations:** 1 Department of Biochemistry, Indian Institute of Science, Bangalore 560 012, India

**Keywords:** HSBP, HSF, Malaria, *Plasmodium falciparum*, Heat shock response

## Abstract

**Background:**

Heat shock factor binding protein (HSBP) was originally discovered in a yeast two-hybrid screen as an interacting partner of heat shock factor (HSF). It appears to be conserved in all eukaryotes studied so far, with yeast being the only exception. Cell biological analysis of HSBP in mammals suggests its role as a negative regulator of heat shock response as it appears to interact with HSF only during the recovery phase following exposure to heat stress. While the identification of HSF in the malaria parasite is still eluding biologists, this study for the first time, reports the presence of a homologue of HSBP in *Plasmodium falciparum.*

**Methods:**

*Pf*HSBP was cloned and purified as his-tag fusion protein. CD (Circular dichroism) spectroscopy was performed to predict the secondary structure. Immunoblots and immunofluorescence approaches were used to study expression and localization of HSBP in *P. falciparum.* Cellular fractionation was performed to examine subcellular distribution of *Pf*HSBP. Immunoprecipitation was carried out to identify HSBP interacting partner in *P. falciparum.*

**Results:**

*Pf*HSBP is a conserved protein with a high helical content and has a propensity to form homo-oligomers. *Pf*HSBP was cloned, expressed and purified. The *in vivo* protein expression profile shows maximal expression in trophozoites. The protein was found to exist in oligomeric form as trimer and hexamer. *Pf*HSBP is predominantly localized in the parasite cytosol, however, upon heat shock, it translocates to the nucleus. This study also reports the interaction of *Pf*HSBP with *Pf*HSP70-1 in the cytoplasm of the parasite.

**Conclusions:**

This study emphasizes the structural and biochemical conservation of *Pf*HSBP with its mammalian counterpart and highlights its potential role in regulation of heat shock response in the malaria parasite. Analysis of HSBP may be an important step towards identification of the transcription factor regulating the heat shock response in *P. falciparum*.

## Background

The human malaria parasite, *Plasmodium falciparum* is exposed to wide ranges of temperature fluctuation during its life cycle. During transmission from the arthropod vector to the human host, it encounters around 12˚C switch in the environmental temperature. Moreover, the parasite has to adapt to temperature fluctuations due to the febrile episodes that occur during clinical manifestation of the disease. Considering the repeated heat stress conditions encountered by the parasite during its life cycle, presence of a robust heat shock response machinery is essential for its survival.

In eukaryotes, there are three main factors regulating heat shock response: (i) heat shock factors (HSF), which are transcription factors regulating heat shock protein (*hsp*) genes; (ii) heat shock elements (HSE), DNA binding motif for HSF binding, present upstream of heat inducible genes; and, (iii) heat shock proteins (HSP), which protect other cellular proteins and also help in the regulation of HSF [1]. The regulation of heat shock response is also supported by certain accessory factors such as heat shock factor binding protein (HSBP), which are known to be involved in attenuation of the heat shock response. While *P. falciparum* is endowed with the presence of a repertoire of HSPs which play a critical role in the life cycle of the parasite, the mechanism of their induction is only partly understood [2-5]. For example, the heat shock transcription factor is yet to be identified in the parasite. Despite the seeming absence of HSF, we show here that *P. falciparum* possesses an HSBP, a known negative regulator of HSF.

Under stress conditions such as heat shock, HSF undergoes transition from monomer to active functional phosphorylated trimer and consequently leads to the induction of HSPs [6]. Attenuation of heat shock response is believed to be mediated by binding of HSBP. In order to attenuate the heat shock response, HSF dissociates from active trimeric form to monomers and thus loses its DNA-binding activity. This shift in oligomeric status is brought about by binding of HSBP and HSP70 to HSF. In initial phase of attenuation, HSBP undergoes transition from hexameric to trimeric form and binds to active trimer of HSF and thus negatively regulates its activity [1,6,7]. Thereafter, HSBP also associates with HSP70 [6]. However, the precise roles of HSBP and Hsp70 in heat shock response remain obscure.

HSBP was first identified in a yeast two-hybrid screen using HSF as the bait [6]. The HSBP domain is highly conserved across all species. The protein has been identified in all species except the budding yeast, *Saccharomyces cerevisiae*[1]. Moreover, in plants, HSBP has also been implicated in seed and endosperm development and embryogenesis [8-10].

The HSBP family of proteins is highly conserved and contains hydrophobic heptad repeats characteristic of coiled-coil proteins and also self-associates leading to oligomerization. Changes in protein oligomerization status are known to be associated with regulation of various cellular processes [7]. This particular phenomenon plays an important role in HSBP-HSF interactions [1]. HSBP is usually localized in the nucleus [1,6,7]. Hsu and colleagues have reported that *Arabidopsis thaliana* HSBP localizes to the cytoplasm and translocates to the nucleus to participate in attenuation of heat shock response [9].

Despite the critical role of heat shock response in life cycle of malaria parasite, regulation of heat shock response in *Plasmodium* is poorly understood. Therefore, studies on *Pf*HSBP in absence of a canonical HSF can provide new insights into our understanding of mechanism of heat shock response in *Plasmodium.* In this study, characterization of *Pf*HSBP was performed to gain insights into its functional roles. *Pf*HSBP (PF3D7_1120900/PF11_0216) is an evolutionarily conserved protein with a high helical content. It shows 28% identity to human HSBP1 protein with the presence of characteristic HSBP core domain. This protein was found to be present as a homo-oligomer in *P. falciparum* and translocates to nucleus upon heat shock. The study also shows that *Pf*HSBP is capable of interacting with *Pf*HSP70-1. These results suggest that *Pf*HSBP is an integral part of heat shock response machinery of *P. falciparum* and its study will address the gap in our understanding of heat shock response in this parasite.

## Methods

### *Plasmodium falciparum* cultures

*Plasmodium falciparum* 3D7 strain was cultured in human O^+^ erythrocytes at 5% haematocrit in RPMI 1640 medium supplemented with 200 μM hypoxanthine, 0.2% (w/v) sodium bicarbonate, 0.2% (w/v) glucose and 0.5% (w/v) Albumax II. For stage-specific studies, parasites were tightly synchronized by 5% sorbitol treatment as described previously and isolated at ring (2–12 hours post infection/hpi), trophozoite (24–30 hpi) and schizont (36–48 hpi) stages. Control *P. falciparum* cultures were grown at 37°C. For heat shock, *P. falciparum* cultures were incubated at 42°C for one hour.

### Bacterial strains, plasmids and growth conditions

*Escherichia coli* strains DH5α and BL21 (DE3) pLysS were cultured at 37°C in Luria broth. Recombinant strains were also cultured under similar conditions with appropriate antibiotics (ampicillin-100 μg/ml and chloramphenicol -34 μg/ml). The plasmid pRSETA was used for expression studies of the *hsbp* gene.

### Antibodies

α-His-tag and α-histone antibodies were commercially purchased. α-His-tag antibody was used at 1:10,000 dilution; α-histone antibody was used at dilution of 1:500 and Horse radish peroxidase conjugated secondary antibody was used at 1:5,000 dilution for western blotting. α-*Pf*Hsp60 antibody was used in dilution 1:1,000. α-*Pf*HSBP polyclonal antiserum was raised in rat against the peptide ‘LSDNLLNKVDNMEKYLDELE’ from *Pf*HSBP sequence. α-*Pf*HSBP antibody was used at 1:500 dilution and anti-rat secondary antibody was used at 1:3,000 dilution for western blotting. For IFA, α-*Pf*HSBP antibody was used at 1:50 dilution and FITC conjugated anti-rat secondary antibody was used at 1:300 dilution. Animal handling was done adhering to the guidelines for animal handling at the Indian Institute of Science.

### Sequence analysis

HSBP protein sequences were downloaded from NCBI database and aligned using MUSCLE algorithm in MEGA 6 suite. A phylogenetic analysis using aligned sequences was performed by the neighbor joining algorithm in MEGA 6.0 software. Bootstrap test with 500 replicates was performed and all positions containing gaps were deleted [11,12]. Secondary structures of *Pf*HSBP and *Hs*HSBP were predicted using Coils [13], which calculates the probability of a structure to adopt a coiled-coil conformation. A three-dimensional model of the *Pf*HSBP was constructed by homology modelling. The human HSBP model prediction was performed using the I-TASSER (iterative threading assembly refinement) server online [14]. The PyMOL program (The PyMOL Molecular Graphics System, Version 1.5.0.4 Schrödinger, LLC) was used to generate the figure for the model structure.

### Cloning and purification of *hsbp* gene

Amplification of *hsbp* gene was carried out using primers *Pf*HSBP_BamHI_F (5′-CCGCG*GGATCC*ATGAATTTTAACGAAATGTTAAAGAG-3′) and *Pf*HSBP_SacI_R (5′-CCGCG*GAGCTC*TTACTGTAAATTATTTTGAGTTGTATAG-3′). The obtained 342 bp product was cloned into pRSETA (BamHI and SacI sites) and transformed into DH5α. To obtain higher expression the recombinant plasmid was transformed in *E. coli* BL21 (DE3) pLysS strain. The protein was over-expressed by induction with 0.5 mom IPTG for 16 hours at 16°C. The culture was lysed by sonication in 6 M Urea, 50 mM Tris-Cl (pH 7.5), 500 mM NaCl, 10% glycerol and 5 mM imidazole with appropriate protease inhibitors. His-tagged *Pf*HSBP was purified using nickel-nitrilotriacetic acid affinity chromatography. Protein was renatured by step-wise dialysis in buffer containing no urea.

### Two-dimensional electrophoresis

Protein was acetone precipitated and dissolved in 2D lysis buffer (7 M urea, 2 M thiourea, 2% CHAPS, 2% ampholytes (pH 3 to 10) and 5% DTT). Isoelectric focusing (IEF) gel was polymerized in tube (7 cm x 1.5 mm) followed by pre-focusing of IEF tube gel at 250 V for 30 min. Protein was then loaded onto tube gel and resolved at 500 V for four hours. Tube gels were incubated in equilibration buffer (125 mM Tris-Cl, (pH 8.8), 2% SDS, 5 mM DTT and 10% glycerol) for 20 min. Second dimension was carried out on 12% SDS-PAGE gel.

### Immunoblotting

The samples were resolved on SDS-PAGE and blotted on nitrocellulose membranes. Immunoblot was performed. The blots probed with HRP-conjugated secondary antibody were developed by chemiluminiscence, whereas blots probed with non-HRP conjugated antibody were developed by nitro-blue tetrazolium chloride (NBT)/5-bromo-4-chloro-3′-indolyphosphate p-toluidine salt (BCIP) method.

### Circular dichroism

Circular dichroism (CD) spectrum was recorded on a Jasco J-810 spectropolarimeter. *Pf*HSBP (in buffer with 50 mM Tris-Cl pH 7.5 and 75 mM NaCl) was scanned from 240 to 200 nm at a scan rate of 50 nm/min. Data were corrected for the baseline with respect to buffer and analysed by program K2D2, which estimates protein secondary structure from CD spectra [15].

### In-gel digestion and mass spectrometry

A narrow slice corresponding to HSBP size was cut from the stained SDS-PAGE gel and further sliced into smaller gel pieces. After several washes with 100 mM ammonium bicarbonate (NH_4_HCO_3_) in 50% acetonitrile, the gel pieces were subjected to a reduction step using 10 mM dithiothreitol in 100 mM NH_4_HCO_3_ buffer (45 min at 56°C). Alkylation was performed with a solution of 55 mM iodoacetamide in 100 mM NH_4_HCO_3_ (30 min at room temperature in the dark) followed by in-gel digestion with 20 μl of trypsin (10 ng/μl) in 50 mM NH_4_HCO_3_ (overnight at 37°C). Subsequently, the peptides were extracted in NH_4_HCO_3_ buffer with 5% formic acid. Samples were vacuum-dried and reconstituted in buffer with 5% formic acid. The protein digest spectrum was acquired on a Q-STAR Elite (QTOF) mass spectrometer equipped with Applied Biosystems Nano Spray II ion source. For identification of proteins, the processed data were searched against NCBI non-reduntant database using the Protein Pilot 4.0 software (threshold 10%) with precursor and fragment mass tolerances of 0.15 Da, cysteine carbamidomethylation as fixed modification and methionine oxidation as variable modification.

### Metabolic labelling and immunoprecipitation

*Plasmodium falciparum* in culture was labelled metabolically with [^35^S] cysteine and -methionine at 150 μCi/ml (BRIT) culture for 12 hours. *Plasmodium falciparum*-infected erythrocytes were lysed with 0.05% saponin (saponin lysis) to obtain the parasites (saponin pellet (SP)), which were lysed with 1% Triton X-100 and the obtained lysate was used for immunoprecipitations (IPs). α-*Pf*HSBP antisera was used at 1:25 dilution and incubated at 4°C for 12 hours on an end-to-end rotator. Protein G beads were then added and incubated for three hours, at the end of which the beads were washed five times (20 min) with wash buffer (1% Triton X-100 in PBS). After the washes, the immunoprecipitates were eluted by boiling in Laemmli buffer and analysed by SDS-PAGE. The gels were vacuum-dried and exposed in a phosphoimager cassette. The film was scanned after 48 hours using a phosphoimager (GE Healthcare).

### Cell fractionation

Cells were lysed (saponin lysis) to obtain parasites. Cytoplasmic and nuclear fractionation was carried out using Lanzeret *et al.* method [16]. Briefly, SP was resuspended in lysis buffer (20 mM HEPES (pH 7.8), 10 mM KCl, 1 mM EDTA, 1 mM EGTA, 1 mM DTT, 0.65% NP-40 and protease inhibitors) and incubated on ice for 5 min. After centrifugation at 2,000 *g* for 10 min, the supernatant obtained is the cytoplasmic extract. The pellet was resuspended in nuclear extraction buffer (20 mM HEPES (pH 7.8), 400 mM NaCl, 1 mM EDTA, 1 mM EGTA, 1 mM DTT, 0.65% NP-40 and protease inhibitors) and vortexed for 10 min. After centrifugation, the supernatant obtained is the nuclear extract.

### Indirect immunofluorescence assay

Immunofluorescence assay was performed as previously described by Tonkin *et al.*[17]. Briefly, cells were washed once with phosphate buffered saline (PBS) and then fixed with 4% paraformaldehyde and 0.0075% glutaraldehyde in PBS for 30 min. Fixed cells were washed once with PBS and then permeabilized with 0.1% Triton X-100 in PBS for 2 min. Cells were then incubated in blocking solution (3% BSA in PBS) for one hour. α-HSBP antibody (1:50 dilution; 3% BSA in PBS) was added and allowed to bind for one hour. Cells were washed with PBS for three times (10 min each) to remove excess primary antibody. FITC-conjugated anti-rat secondary antibody (1:300 dilution; 3% BSA in PBS) was added and allowed to bind for one hour. Cells were washed three times with PBS and mounted in 70% glycerol with 2% DABCO. The coverslips were then inverted onto a glass microscope slide, mounted and sealed.

### Gel permeation chromatography

The size exclusion chromatography was carried in Superdex 200 (10/300 GL column; GE Healthcare) using NGC Chromatography system (Bio-Rad). Column Washes and elution of sample was carried out in PBS buffer.

## Results

### *Pf*HSBP is distinct amongst other conserved HSBPs

**Figure 1:**
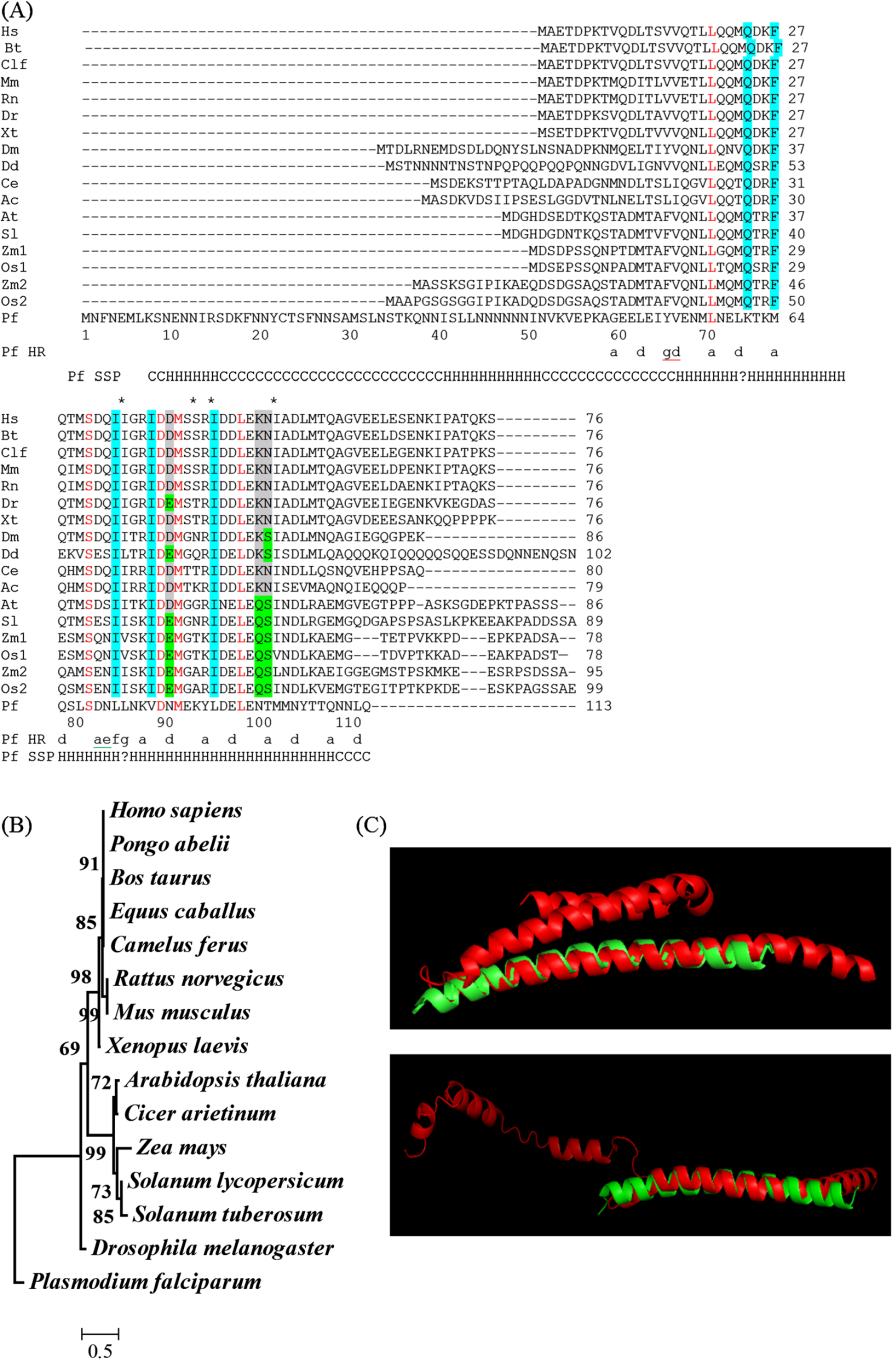
**Bioinformatics analysis of *Pf*HSBP. (A)** Sequence alignment of *Pf*HSBP with related HSBPs from other organisms. **(B)** Phylogenetic tree for HSBP, shows *Pf*HSBP is an ancestral protein to both plants and animal HSBPs. Optimal tree with sum of branch length = 2.72 is shown and bootstrap value is shown next to the branch. **(C)***Pf*HSBP models (Red), predicted using I-TASSER server online, aligned with *Hs*HSBP (green).

*Pf*HSBP (PF11_0216), a 113-residue protein, is comprised mainly of α-helix along with coiled-coil segments. The longest helical segment (residues 62–106 of *Pf*HSBP corresponding to 11–55 of *Hs*HSBP) is the most conserved region across all species. *Pf*HSBP has an additional N-terminus region (residues 1–60). A sequence alignment with other HSBPs is shown in Figure 1A. For correlation, residue 52 of *Pf*HSBP aligns with residue 1 of *Hs*HSBP. Residues 71, 82, 90, 92, and 99 (corresponding to 20, 31, 39, 41, and 48 in *Hs*HSBP) are conserved across all species (shown in red in Figure 1A). Ser82 is conserved across all species and probably plays an essential role in biological function [1]. Residues 75, 78, 85, 89 and 96 in *P. falciparum* are distinct. Corresponding residues are identical in all other species (shown as blue shade in Figure 1A). Residues 91, 101 and 102 in *P. falciparum* are also distinct. However, corresponding residues in all other species are conserved (shown as silver/green shade in Figure 1A).

The *Pf*HSBP protein has ambiguity in heptad repeats at two positions ((i) residue 67 - ‘abc’ is missing; and, (ii) residue 86 -‘bcd’ is missing). These stutters are in the N-terminus region of the conserved portion. Whereas, the C-terminus of the protein (residues 89–113 of *Pf*HSBP corresponding to 38–62 of *Hs*HSBP) comprises of continuous heptad repeats (Figure 1A). Helix probability of *Pf* HSBP and *Hs*HSBP sequences was calculated using prediction program, Coils [13]. *Pf*HSBP has longer helix compared to human homologue. The low helix propensity region at two positions (residues 67 and 86) within *Pf*HSBP domain coincides with the positions of stutters in heptad repeats.

Phylogenetic tree of HSBP (Figure 1B) shows that within animal and plant kingdoms, HSBP homologues are very similar and are clustered together. Conversely, *Pf*HSBP is very distant and is ancestor to both the clusters. The *Pf*HSBP (red) structure, predicted using I-TASSER server online, when aligned with *Hs*HSBP (green) showed that the structural pattern of HSBP domain was similar (Figure 1C) [14].

Oligomerization propensity for *Pf*HSBP and *Hs*HSBP was calculated using prediction program, Multicoil [18]. It is well documented that *Hs*HSBP forms a trimer [1,6,7]. However, the probability of trimerization is higher in *Pf*HSBP compared to *Hs*HSBP. The probability of dimer: trimer is 1:3 for *Pf*HSBP, whereas, the same for *Hs*HSBP is only 1.5:1. With respect to total probability, 75% of *Pf*HSBP can form trimer, whereas only 40% of *Hs*HSBP can form trimer.

### *In vitro* characterization of *Pf*HSBP

**Figure 2:**
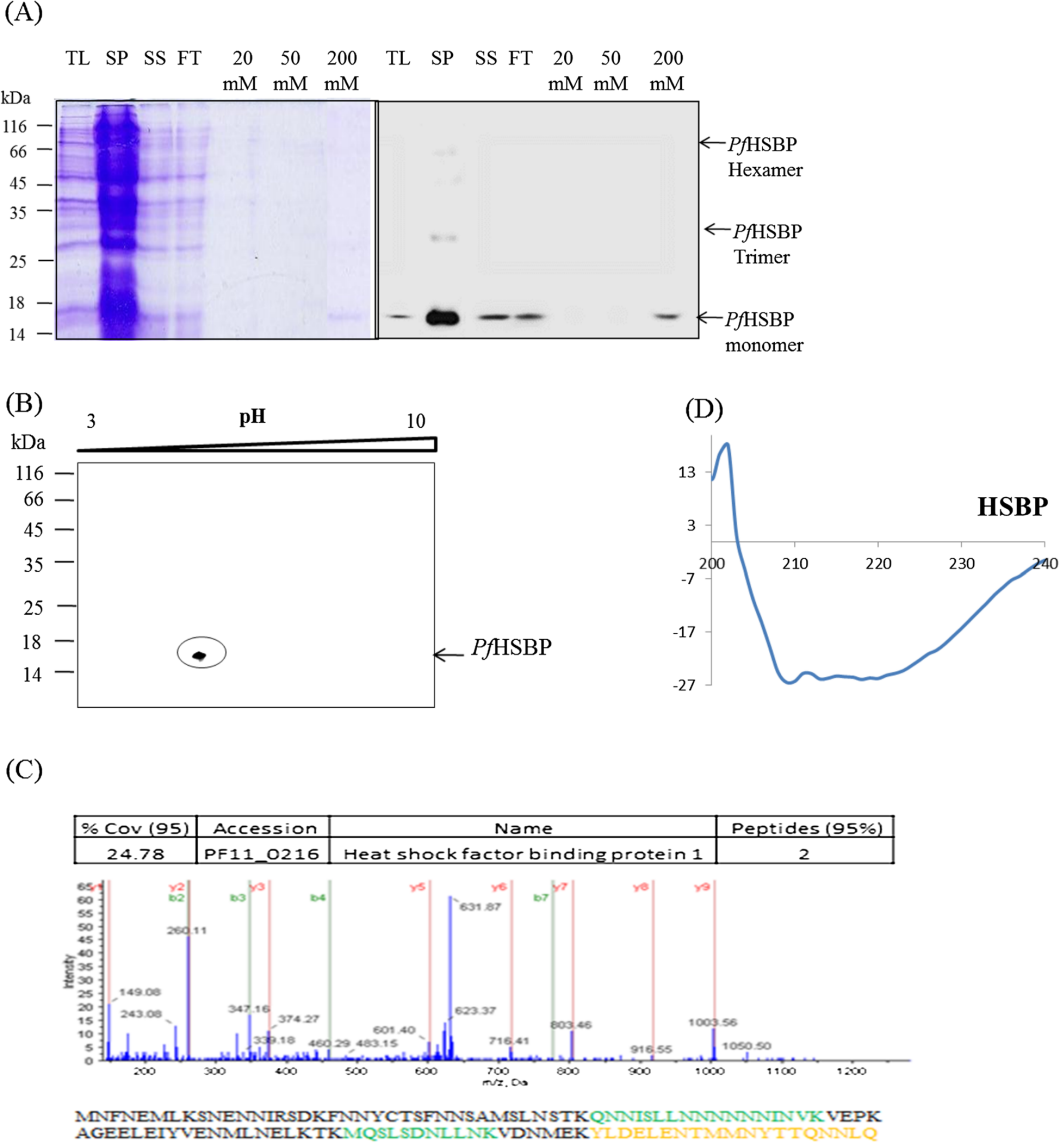
***In vitro *characterization of *Pf*HSBP. (A)** 15% SDS-PAGE gel and immunoblot showing purified recombinant *Pf*HSBP. Lanes:- TL - total lysate, SP - pellet remaining after sonication, SS -supernatant after sonication, FT -flow through, 20 mM - wash with 20 mM imidazole, 50 mM - wash with 50 mM imidazole, and 200 mM - elution with 200 mM imidazole. **(B)** 2-D-electrophoresis of purified recombinant protein. **(C)** MS-based identification of *Pf*HSBP. **(D)** Circular dichroism spectrum of *Pf*HSBP.

*Pf*HSBP was cloned from *P. falciparum* cDNA in pRSET-A vector as a 6x- his tagged fusion protein and confirmed by insert release by double digestion by BamHI and SacI. This fusion protein was expressed in *Escherichia coli* BL21 pLysS and was purified to homogeneity using Ni-NTA chromatography (Figure 2A). Identity of the protein was confirmed by performing immunoblot using an α-His tag antibody. A band corresponding to his tagged *Pf*HSBP was observed at 17 kDa (Figure 2A). Two-dimensional electrophoresis was carried out for the purified protein. Isoelectric point of purified protein was observed to be ~4 which corresponds to its theoretical pI, 4.37 (Figure 2B).

To validate the identity of the protein, MS/MS analysis of purified *Pf*HSBP was carried out (Figure 2C). Homology-driven searches for protein identification from MS/MS data in *Plasmodium* database by using Protein Pilot, identified the protein as *Pf*HSBP. Peptide scores above 50 and a protein score of 1.3 corresponding to a confidence level greater than 95% were used with error tolerance of 100 ppm.

In order to analyse the secondary structure of *Pf*HSBP, circular dichroism (CD) studies were carried out with purified recombinant *Pf*HSBP. Knowing the important functional role played by the helical segment, it was important to perform this experiment in order to draw parallels with the known secondary structures of other HSBPs. In addition, the extra N-terminal stretch possessed uniquely by *P. falciparum*, could contribute to changes in the secondary structure of the protein. The CD spectrum (Figure 2D) showed double minima at 208 and 222 nm characteristic of helical proteins. Spectrum analysis using K2D2 software showed a high helical content (85%) [15].

### *Pf*HSBP is expressed maximally in trophozoites and exists as an oligomer

**Figure 3:**
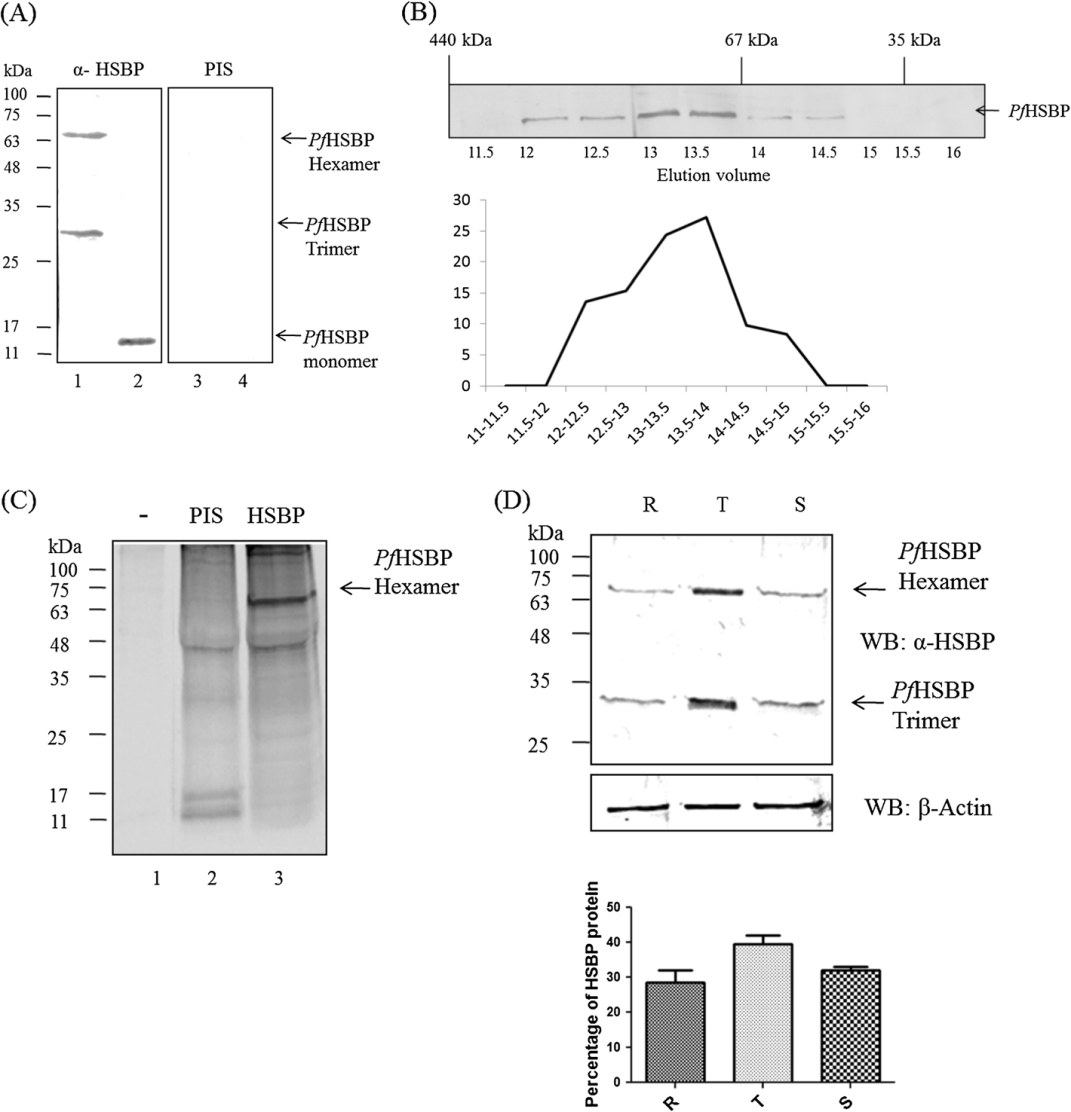
***In vivo Pf*HSBP expression. (A) ***Pf*HSBP exists in *P. falciparum* as a trimer and hexamer (Lanes 1 and 3 -*P. falciparum* lysate; lanes 2 and 4 - purified recombinant protein. Lanes 1 and 2 were probed with polyclonal antibody generated against *Pf*HSBP, whereas, lanes 3 and 4 were probed with pre-immune serum). **(B)** Size exclusion chromatography of *Pf*HSBP. Immunoblot shows the fractions of *P. falciparum* lysate containing *Pf*HSBP (top panel). The graph represents quantitation of *Pf*HSBP signal obtained (bottom panel). **(C)** Immunoprecipitation by *Pf*HSBP antiserum shows pull down of hexameric *Pf*HSBP, Lane 1-Protein-G control, Lane 2- Pre immune serum control, Lane 3- *Pf*HSBP IP. **(D)***Pf*HSBP expression pattern in different blood stages of *P. falciparum*. (R - Ring stage, T - Trophozoite stage, S - Schizont stage). β-actin was used as a loading control. Bar diagram shows quantitative amounts of *Pf*HSBP present in different stages of *P. falciparum.*

In order to check the expression status of *Pf*HSBP in the parasites, the *Pf*HSBP antiserum was probed against both *P. falciparum* lysate and the recombinant protein (Figure 3A). The anti-*Pf*HSBP anti-serum reacted specifically with multimers of *Pf*HSBP in *P. falciparum* lysate (lane 1) and the purified protein (lane 2). The pre-immune serum does not react with the *Pf*HSBP in *P. falciparum* lysate (lane 3) and the purified protein (lane 4). Interestingly, the recombinant *Pf*HSBP exists in monomeric form, whereas *in vivo Pf*HSBP exists in the form of trimer and hexamer. The *Pf*HSBP oligomer is Sodium dodecyl sulphate-resistant. Moreover, the protein has an anomalous mobility as it does not run at its corresponding molecular weight. The molecular weight of *Pf*HSBP monomer, trimer and hexamer corresponds to 13 kDa, 39 kDa and 78 kDa, respectively. However, on SDS-PAGE, the trimeric and hexameric forms run approximately at sizes 30 kDa and 70 kDa, respectively. The size exclusion chromatography (Figure 3B) performed on *P. falciparum* lysate also showed that *Pf*HSBP *in vivo* exists as hexamers (~70 kDa). Tai and colleagues had concluded similar facts for human *Pf*HSBP based on gel filtration and analytical ultracentrifugation studies [7]. Immunoprecipitation by HSBP antiserum showed presence of *Pf*HSBP (hexamer; ~70 kDa) in *P. falciparum* lysate (Figure 3C).

The *Pf*HSBP gene expression pattern obtained from PlasmoDB shows that the maximum expression occurs in the trophozoite stage indicated by the peak during 20–32 hours post invasion (hpi) [19]. To examine whether the transcription profile correlates with its proteomic expression, immunoblot analysis was carried out for *Pf*HSBP for the three intra-erythrocytic stages of *P. falciparum* (Figure 3D). The immunoblot depicting the relative expression of *Pf*HSBP (normalized by actin) also clearly showed that the protein was maximally expressed in the trophozoite stages followed by rings and schizont. As mentioned previously, *Pf*HSBP exists as a multimer *in vivo*. It is expressed as trimeric as well as hexameric form in all the three stages of the parasite’s life cycle.

### *Pf*HSBP translocates to the nucleus upon heat shock

**Figure 4:**
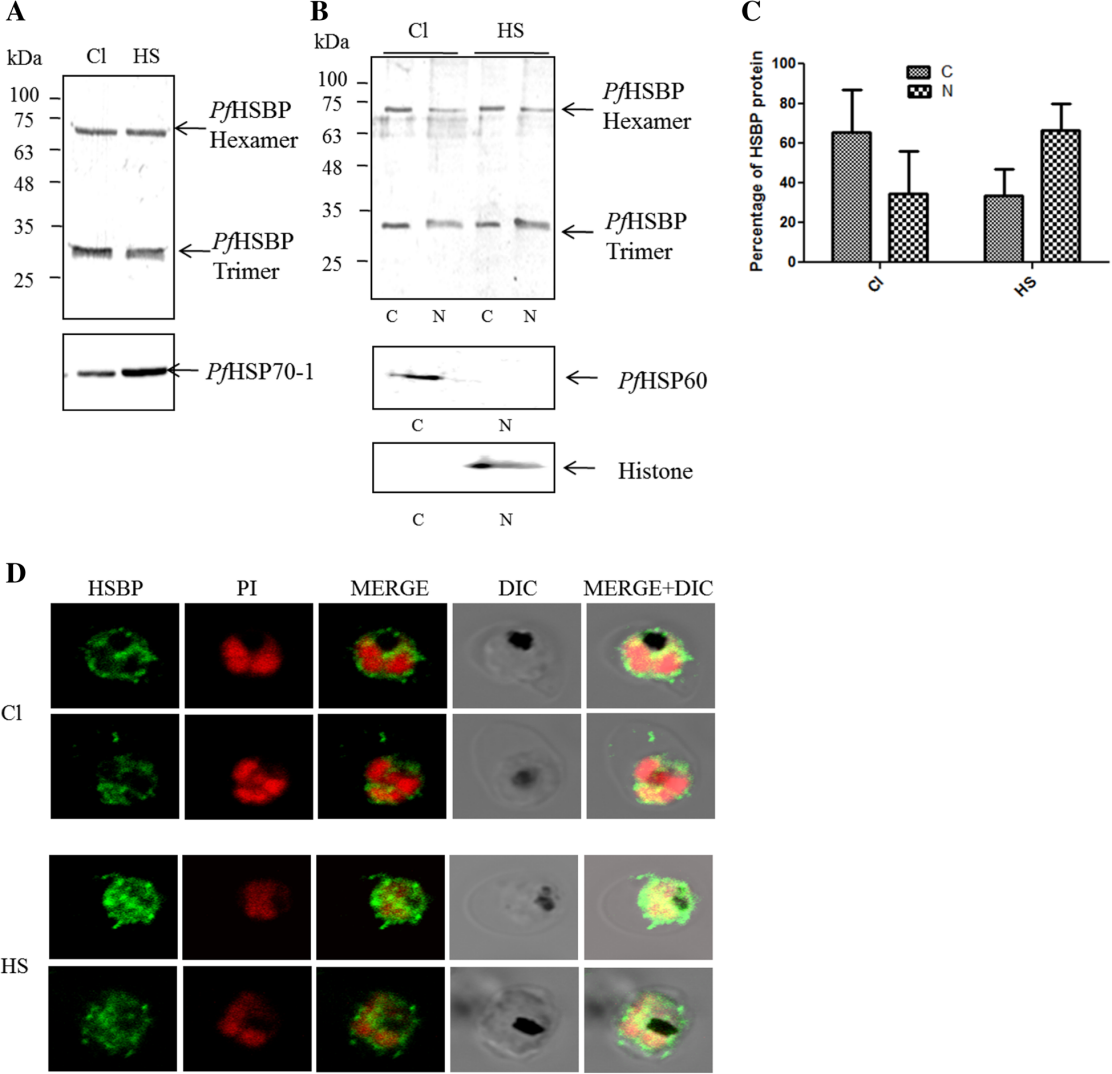
***Pf*HSBP undergoes nuclear translocation upon heat shock. (A)** immunoblot analysis to examine *Pf*HSBP expression under normal and heat shock conditions reveals that *Pf*HSBP does not get heat induced; **(B)** immunoblot analysis of cytoplasmic and nuclear fractions to determine the localization of *Pf*HSBP under normal and heat shock conditions; **(C)** relative amount of *Pf*HSBP in cytoplasmic and nuclear compartments in normal and heat shock conditions. Upon heat shock amount of *Pf*HSBP in nucleus increases; **(D)** IFA to determine the localization of *Pf*HSBP under normal and heat shock conditions. (Cl - Control condition (37°C), HS - heat shock condition (42°C); C - cytoplasm, N - nucleus) reveals nuclear translocation of *Pf*HSBP upon heat shock.

HSBP is known to get upregulated in plants upon heat shock [9,10]. In order to examine whether *Pf*HSBP gets heat-induced, immunoblot analysis was performed. The parasite culture was heat shocked at 41°C for one hour. The amount of total *Pf*HSBP did not increase under heat shock stress compared to normal conditions (Figure 4A). *Pf*HSP70-1 (known to be induced on heat shock) was taken as a control. In *P. falciparum,* where HSF remains yet unidentified, the localization studies of *Pf*HSBP can provide insights about its role in heat shock response. *Plasmodium falciparum* cells were fractionated in order to obtain nuclear and cytoplasmic fractions. Both the fractions were found to possess *Pf*HSBP when probed with α-*Pf*HSBP antiserum (Figure 4B - lanes Cl C (cytoplasm) and Cl N (nucleus)). Upon quantitation, about 65% of total *Pf*HSBP was found to be localized in cytoplasmic fraction (Figure 4C). The cells were subjected to heat shock to look for a differential localization and/or oligomerization pattern, if any. However, upon heat shock, a change in distribution of the protein was observed. Sixty to seventy percent of *Pf*HSBP could now be detected in the nuclear fraction (Figure 4B - lanes HS C (cytoplasm) and HS N (nucleus), Figure 4C). α-*Pf*HSP60 and α-Histone known to localize in cytoplasm and nucleus, respectively, were used as fractionation controls.

The localization of *Pf*HSBP in the parasite was further confirmed by indirect immunofluorescence analysis (IFA). The signal for *Pf*HSBP in control cells can be seen majorly in cytoplasm and a small fraction inside the nucleus (Figure 4D (Cl)). On subjecting the cells to heat shock, *Pf*HSBP was found to be localized in both the compartments (Figure 4D (HS)). However, the relative amount in nucleus increases significantly. These results clearly suggest that despite absence of a nuclear localization signal, *Pf*HSBP undergoes nuclear translocation upon heat shock and thereby pointing out its involvement in the heat shock response.

### *Pf*HSBP interacts with HSP70-1

**Figure 5:**
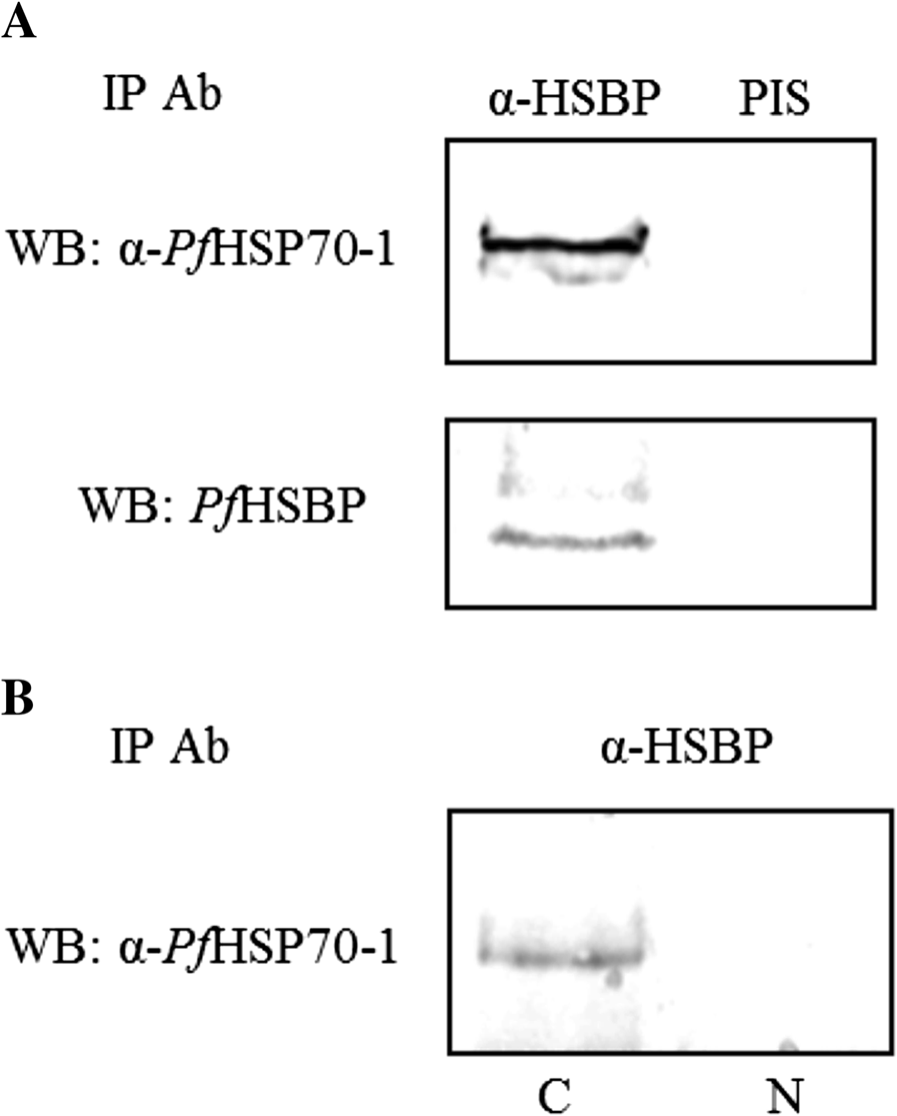
**Co-immunoprecipitation with α-*Pf*HSBP antiserum confirms *Pf*HSBP-*Pf*Hsp70 interaction. (A)** immunoprecipitation performed on *P. falciparum* lysate using α-HSBP antibody followed by immunoblot analysis with α-*Pf*HSP70-1 and α-*Pf*HSBP antibody. Signal for *Pf*HSP70-1 in the immunoblot indicates interaction between the two proteins; **(B)** immunoprecipitation performed on nuclear and cytoplasmic fractions of *P. falciparum* using α-*Pf*HSBP followed by α-*Pf*HSP70-1 immunoblot. *Pf*HSP70-1 signal is obtained in the cytoplasmic fraction.

In other biological systems, HSBP participates in heat shock response attenuation by binding, destabilizing and dissociating HSF trimers to the inert monomers, in conjunction with HSP70. HSBP is also reported to interact with HSP70 during the attenuation phase of heat shock response. Also, it is known that HSP70 is induced upon exposure to stress such as heat shock. Therefore, it was of interest to look at the association between HSBP and HSP70 in the malaria parasite. In order to examine whether *Pf*HSBP and *Pf*HSP70 interact with each other, co-IP was performed with α-*Pf*HSBP antibody. The immunoprecipitate was subjected to immunoblot with α-*Pf*HSP70-1. A clear signal for *Pf*HSP70-1 was detected, which was not observed in the pre-immune serum (PIS) control. The immunoblot was also probed with the IP antibody, α-*Pf*HSBP (Figure 5) as a control (Figure 5A).

Having seen that *Pf*HSBP and *Pf*HSP70 are present in a common complex, it was of interest to map the localization of this interaction. To this end, nuclear fractionation was carried out to obtain nuclear and cytoplasmic fractions. Immunoprecipitation was thereafter performed on both these fractions with α-*Pf*HSBP antibody. It can be observed that in *P. falciparum*, *Pf*HSBP and *Pf*HSP70 interaction takes place in the cytoplasmic compartment (Figure 5B).

## Discussion

The canonical pathway of heat shock response in eukaryotes involves a heat shock transcription factor serving as a sensor of heat shock in the cytoplasm. On exposure to heat shock, HSF undergoes trimerization-based activation and translocates to the nucleus. Such activated HSF binds specifically to HSE in the promoter region of hsp genes and induces their transcription.

In addition to the above core components of the heat shock response machinery, a heat shock factor binding protein (HSBP) has been implicated in the regulation of the heat shock response. HSBP was shown to bind and negatively regulate HSF in mammalian cells [1,6]. Under conditions of stress, HSBP exists in an inactive state but during recovery from stress it associates with HSF and Hsp70 presumably involved in attenuation of the heat shock response [1].

Despite the relevance of heat shock response in the pathogenesis of malaria, the HSF necessary for up regulating the transcription of hsp genes has not been identified as yet. However other transcription factors belonging to the AP2 family have been implicated in the up regulation of hsp genes [20,21]. Despite the absence of a classical HSF, malaria parasite does seem to possess a homologue of HSBP.

HSBP are conserved proteins (<10 kDa) with a high helical content. Interestingly, *Pf*HSBP is longest (13.17 kDa) amongst all HSBP studied thus far by virtue of an extra N-terminal region. The *Pf*HSBP domain completely aligned with its human counterpart and thus suggesting similar protein function across species. Bioinformatic analysis suggests that the extra N-terminal segment does not correspond to any known domain. *Pf*HSBP appears to be phylogenetically distant from its animal or plant counterparts. This could be attributed to the extra N-terminal region possessed by *Pf*HSBP. The recombinant protein predominantly exists in monomeric form. Like HSBP from other organisms *Pf*HSBP also exhibits anomalous mobility on SDS-PAGE [7].

Studies carried out on HSBP from mammalian and plant systems have revealed its functional oligomerization. This protein is known to oligomerize *in vivo* into trimers and hexamers. The hexameric form of the protein gets converted into the active trimeric form which in turn interacts with the HSF trimer during the attenuation of heat shock response. *In vivo, Pf*HSBP exist as trimer as well as hexamer and these multimers were detergent resistant. *Pf*HSBP was maximally expressed in trophozoite stage of the parasite. *Pf*HSBP predominantly localized in the cytoplasm under normal conditions, however, upon heat stress, it translocated to the nucleus. Nuclear translocation has also been observed in *Arabidopsis thaliana*[9]. Nuclear localization of *Pf*HSBP upon heat shock is suggestive of its potential role in regulation of heat shock response.

Studies on HSBP have implicated its role as a negative regulator of HSF [6]. The association of HSBP with HSF and HSP70 coincides with attenuation of heat shock response mechanism and conversion of HSF to inactive form [6]. HSBP initially interacts with HSF and then with HSP70 during recovery from heat shock. However, the precise mechanism remains unclear. This study shows that *Pf*HSBP is also capable of interacting with *Pf*HSP70-1 in the cytoplasm.

This study, for the first time, reports the presence of a HSF binding partner, HSBP, in *P. falciparum*. In the absence of a known HSF, this study on *Pf*HSBP serves as a prelude in understanding heat shock response machinery in the malaria parasite.

## Abbreviations

HSBP: Heat shock factor binding protein; HSF: Heat shock factor(s); hpi: Hours post-infection; HSP: Heat shock protein; HSE: Heat shock elements; IEF: Isoelectric focusing; BCIP: 5-bromo-4-chloro-3′-indolyl phosphate; NBT: Nitro-blue tetrazolium; SP: Saponin pellet; IP: Immunoprecipitation; CD: Circular dichroism; IFA: Indirect immunofluorescence assay.

## Competing interests

The authors declare that they have no competing interests.

## Authors’ contributions

SKS, VS, SC, MS, and SV performed all the experiments and analysed data. UT conceived the study. VS, SC, MS, and UT wrote the manuscript. All authors read and approved the final manuscript.
